# Protein Expression of PTTG-1, OCT-4, and KLF-4 in Seminoma: A Pilot Study

**DOI:** 10.3389/fendo.2019.00619

**Published:** 2019-09-11

**Authors:** Giuseppe Grande, Domenico Milardi, Maurizio Martini, Tonia Cenci, Gaetano Gulino, Francesca Mancini, Antonio Bianchi, Alfredo Pontecorvi, Francesco Pierconti

**Affiliations:** ^1^Division of Endocrinology, Fondazione Policlinico Universitario Agostino Gemelli IRCCS, Rome, Italy; ^2^International Scientific Institute Paul VI, Rome, Italy; ^3^Division of Anatomic Pathology and Histology, School of Medicine, Catholic University of Rome, Rome, Italy; ^4^Department of Urology, Fondazione Policlinico Universitario Agostino Gemelli IRCCS, Rome, Italy

**Keywords:** testis cancer, seminoma, PTTG-1, OCT-4, KLF-4, infiltration

## Abstract

Seminomas are the most frequent kind of testicular germ cell tumors (TGCTs), accounting for 50% of tumor diagnosis in young men, whereas non-seminomas account for 40% and mixed forms for 10% of cases. It is currently supposed that TGCTs evolve from a pre-invasive stage of carcinoma *in situ* (CIS). Octamer-binding transcription factor 4 (OCT4) is essential for self-renewal of stem cells. It is considered as a major regulator of cell pluripotency. Prior studies have shown that seminoma expresses OCT4. Transcription factor Krüppel-like factor 4 (KLF4) has moreover associated with embryonic stem cell maintenance. Finally, we previously demonstrated the expression of PTTG1 in CIS and seminomas. In this pilot study, we compared the combined expression of PTTG1 with KLF4 and OCT4 in seminoma, in order to validate our hypotesis that PTTG1 marks a specific population of stem cells in neoplastic tissue, strictly related with tumor. Formalin-fixed and paraffin-embedded testicular tissues by 5 patients who underwent an orchidectomy for seminoma have been collected and immunofluorescence analysis was performed using antibody rabbit monoclonal PTTG-1 and mouse monoclonal OCT4 or mouse monoclonal KLF4 antibody. In seminoma we observed that tumor cells strongly express OCT-4 in all seminomas and in the intratubular areas of seminoma. Expression of KLF-4 was observed in many tumor cells. PTTG1 marks some specific OCT4- and KLF4-positive tumor cells, mainly localized at the periphery of the neoplasm. In the intertubular infiltration areas nests of cells expressing both OCT4/KLF4 and PTTG1 have been observed. This is the first identification of a cell population in seminoma characterized for being OCT4, KLF4, and PTTG1 positive cells in seminoma, associated with cancer invasiveness. Further investigation is needed to elucidate if a functional abrogation of PTTG1 might be used in order to offer new therapeutic approaches in the clinical workout of seminoma.

## Introduction

Seminomas are the most frequent type of testicular germ cell tumors (TGCTs), accounting for 50% of cases in young men, whereas non-seminomas account for 40% and mixed forms for 10% of cases (1). Despite the high prevalence of TGCTs, the molecular mechanisms associated with their development have not been still completely clarified.

It is currently supposed that TGCTs evolve from a pre-invasive stage of carcinoma *in situ* (CIS) (2).

CIS are macroscopically distinct cells that are located on the basement membrane of the seminiferous tubules in the testis and have specific morphological features more similar to embryonic germ cells than spermatogonial stem cells (3). CIS are considered the precursors of seminomas since they both histologically resemble primordial germ cells (PGCs) and gonocytes and have a positive staining for c-kit and PLAP.

For instance, the oncogene c-kit, which encodes for a transmembrane tyrosine kinase receptor, is highly expressed in TGCTs. C-kit has as its specific ligand the stem cell factors and it is required for normal development of germ cells (4, 5). c-kit is highly expressed in seminomas and teratomas (6).

Placental alkaline phosphatase (PLAP) is moreover considered a widely used marker for TGCTs (7).

Apart from the well-known markers (i.e., PLAP and c-kit), previous studies have been carried out to identify new molecular markers for TGCTs.

Octamer-binding transcription factor 4 (OCT4) is a homeobox transcription factor that is essential for self-renewal of stem cells. It is considered as a major regulator of cell pluripotency (8). Importantly, it has been implicated in tumorigenesis of primordial germ cells. Prior studies demonstrated the expression of OCT4 in seminoma (9).

Transcription factor Krüppel-like factor 4 (KLF4) is strongly expressed in postmeiotic spermatids and in Leydig cells, but has been not reported in spermatogonia (10). KLF4 is involved in embryonic stem (ES) cell maintenance (11, 12). Simultaneous depletion of Klf4, Klf2, and Klf5 lead to ES cell differentiation, confirming the critical role of KLF4 in the maintenance of ES cell pluripotency and selfrenewal. Moreover, KLF4 was used, associated with other transcriptional factors, to induce pluripotency in differentiated cells (13). Finally, KLF4 was expressed in mouse spermatogonial stem cells shortly after withdrawal from the stem cell niche (14) in addition to pluripotent cells derived from human testis.

Previous data reported that altered levels of Pituitary-tumor-transforming-gene 1 (PTTG1) are expressed in pre-cancer lesions, suggesting that PTTG1 has a role in human tumorigenesis (15). We previously examined firstly the expression of PTTG1 in CIS and seminomas (16). In CIS, only isolated cells express PTTG1. Furthermore, in the peripheral area of seminoma, PTTG1 was mostly detected as localized in the nucleus, whereas in the central nucleus of seminoma, PTTG1 was mainly expressed in cytoplasm. Moreover, in the zones of seminoma infiltration we demonstrated the presence of clusters of PTTG1-positive cells. We hypotyzed that PTTG1 marks a population of neoplastic cells, both in CIS and in seminoma, so linking CIS to seminoma carcinogenesis. Interestingly, no differences have been observed in the expression of PTTG1 in foci and micronodules of seminoma, so that we hypothesized that when the tumor has a small size, in the early stage of the carcinogenesis, PTTG-1 expression is homogeneously distributed. On the contrary, with the increasing tumor size, this subgroup of nuclear PTTG1-positive cells move from the center to the periphery of the tumor, and it might be associated with neoplastic infiltration of surrounding tissue. PTTG1 in fact is known to play an important role in tumor infiltration and neoplastic angiogenesis. PTTG1 expression in neoplastic cells on the tumor infiltration area and in the intertubular spaces may reflect this property important for tumor cells in invading surrounding tissues and inducing neoplastic angiogenesis.

In this pilot study, we compared the combined expression of PTTG1 with KLF4 and OCT4 in seminoma, in order to validate our hypothesis that PTTG1 could mark a specific subset of neoplastic stem cells, strictly related with tumor.

## Materials and Methods

The study was conducted in accordance with the guidelines of the Declaration of Helsinki. Written informed consent was obtained from each patient.

Formalin-fixed and paraffin-embedded testicular tissues by 5 patients who underwent an orchidectomy for seminoma were collected at the Department of Surgical Pathology Fondazione Policlinico “A. Gemelli” from 2014 to 2017. The age of the patients ranged between 25 and 70 years with a median of 36.

After deparaffinization tissues slides were rehydrated using a graded alcohol solution. Antigen retrieval was performed in 10 mM citrate buffer at pH 6.0 for 10 min in microwave oven. After allowing to cool room temperature, slides were washed twice in distilled water for 2 min and sequentially rinsed once in PBS for 5 min.

To confirm the diagnosis of seminoma we evaluated the co-expression of PLAP and c-kit. For detection of PLAP the monoclonal antibody 8A9 (Dako, Hamburg, Germany; dilution 1:50) was chosen because staining with this antibody is more sensitive compared with other PLAP antibodies.c-KIT was detected by rabbit polyclonal antibody (c-KIT antibody from Dako; dilution 1:100). All primary antibodies were incubated overnight at 4°C. Immunoreactions were visualized by means of the avidin–biotin–complex (ABC method) using AEC (3-amino-9-ethylcarbazol) as chromogen on an immunostainer (Techmate 500; Dako).

Sections have been then incubated a room temperature with primary antibody rabbit monoclonal PTTG-1 (Securin, clone EPR3240, abcam, Cambrige, UK, 1:500 for 1 h). The PTTG-1 were visualized using the highly cross-adsorbed, Alexa Fluor 488-conjugated goat anti-Rabbit IgG secondary antibody (TermoFisher Scientific, USA, 1:1000 for 1 h). After the slides were rinsed in PBS and subsequently were incubated with mouse monoclonal OCT4 (clone OTI9B7, Novus Biological, UK 1:50 for 30 min) or with mouse monoclonal KLF4 (clone CL5785, Novus Biological, 1:1000 for 30 min). This antibodies were visualized using the highly cross-adsorbed, Alexa Flour 594-conjugated goat anti-Mouse IgG secondary antibody (TermoFisher Scientific, 1:1000 for 1 h). Slides are rinsed in PBS, mounted in Vectashield (H-1000, Vector Laboratories, Peterborough, UK) and double immunofluorescence slides were visually examined under immunofluorescence microscopy using an Olympus BX41 fluorescence microscope (Olympus, Although Center, Valley;PA, USA) and digital images were captured using and attached Olympus DP71 digital camera with the x40 objective.

## Results

Clinical and ultrasonographic informations are reported in Table 1.

**Table 1:** Clinical and ultrasound characteristics of the patients.

**Patient *n***.	**Age**	**History of cryptorchidism**	**Infertility**	**Testis size**	**Ultrasound characteristics**	**Tumor size**	**TMN**
1	25	X		10 ml	Hypoecoic single area; testicular microlithiasis	3 × 2 × 2 cm	T1M0N0
2	25			16 ml	Multiple hypocoic areas	2.5 × 3 × 3.5 cm	T2M0N0
3	26		X	20 ml	Hypoechoic single nodule with hyperechoic isles	3 × 2.5 × 3 cm	T2M0N0
4	36	X	X	12 ml	Iso-hypoechoic with hyperechoic striae	2.5 × 1.5 × 2 cm	T1M0N0
5	70	X		10 ml	Hypoecoic single area	2.5 × 2 × 2 cm	T2M0N0

All seminomas demonstrated the co-expression of PLAP and c-kit.

**Figure 1:**
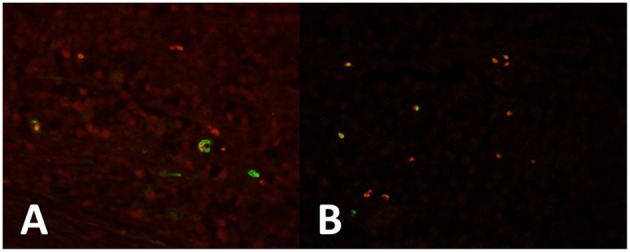
**(A)** Seminoma expresses strong OCT4 expression in all cells (red). PTTG1 marks some specific OCT4-positive (green) tumor cells, mainly localized at the periphery of the neoplasm. **(B)** KLF-4 is expressed in some tumor cells (red). PTTG1 marks some specific KLF4-positive tumor cells (green).

We moreover observed that tumor cells strongly express OCT-4 in all seminoma cells and in the areas of intratubular seminoma (Figure 1A). Expression of KLF-4 was observed in many tumor cells (Figure 1B). PTTG1 marks some specific OCT4-(Figure 1A) and KLF4-positive (Figure 1B) tumor cells, mainly localized at the periphery of the neoplasm.

**Figure 2:**
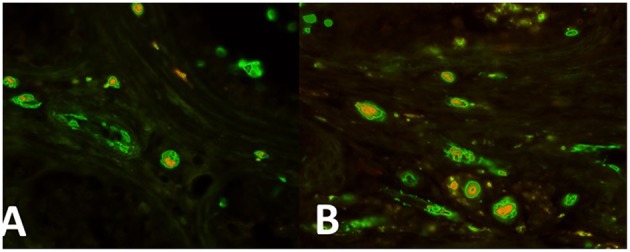
**(A)** Nests of cells expressing both OCT4 (green) and PTTG1 (yellow) in the intertubular infiltration infiltration area. **(B)** Areas nests of cells expressing both KLF4 (green) and PTTG1 (yellow) in the intertubular infiltration infiltration area.

In the intertubular infiltration areas nests of cells expressing both OCT4/KLF4 and PTTG1 (Figures 2A,B, respectively) have been observed.

## Discussion

PTTG1 is a securin protein involved in facilitating sister-chromatid separation in metaphase (17) and exerts a crucial role during the mitosis by defending the chromosomal stability (18). In normal testicular tubules PTTG1 has been identified in normal spermatocytes and spermatids, suggesting that PTTG-1 may play a pivotal function in spermatogenesis in testis, and specifically in differentiation and survival of germ cells (16). PTTG1 in moreover an oncogene since its overexpression induces aneuploidy (19) and stimulates tumor formation, as previously reported in pituitary, thyroid, breast, uterine, ovarian, lung and colon tumors (20–24). In malignant tumors, previous data demonstrated the association between PTTG1 levels, tumor angiogenesis and metastasis (24). Moreover, high expression of PTTG1 is associated with greatly aggressive tumor and with the onset of metastasis (25).

PTTG1 has been demonstrated to contribute to cell migration, invasion and angiogenesis by induction of MMP-2 secretion and expression (26). In lung cancer has been demonstrated the concomitant overexpression of PTTG1 or MMP9. Xu et al. demonstrated that the co-expression of PTTG1 and MMP9 is associated with tumor cell migration and proliferation (27). PTTG1 might also play a pivotal role in inducing tumor angiogenesis in seminoma mainly through the regulation of the expression of many angiogenic factors, including bFGF, VEGF and IL-8 (28–30).

In this study we confirmed that some cells in seminoma express PTTG1 and demonstrated that it is a sub-population of tumor stem cells OCT4- and KLF4- positive cells.

Germ cells, in the early phases of embryonic development, are reserved as primordial germ cells, in order to escape the signals involved in the differentiation of the somatic cells of the embryo (31). Primordial germ cells preserve so their undifferentiated state, as demonstrated by the expression of a lot of stem cell markers, including OCT4, NANOG, stage-specific embryonic antigens and tumor rejection antigens (2, 32). The spermatogonial stem cells (SSC) can moreover be reprogrammed in cell culture to recover a pluripotent state. These findings demonstrate that the SSC are in a relatively primitive developmental state, permitting the reconversion into an early embryonic, pluripotent state without any genetic modification. Molecular studies demonstrated that SSC occasionally functionally go away from the control of their niche, which naturally restricts their developmental potency to normal spermatogenesis and also regulates proliferation. If the stem cell niche fails to control the proliferation of gonocytes or SSC, a transformation of germline stem cells is supposed to occur, thus resulting in a CIS (33, 34). This early neoplasia can subsequently produce a seminoma or an embryonal carcinoma (35).

Furthermore, we have reported in intertubular infiltration areas the presence of nests of cells expressing both OCT4/KLF4 and PTTG1. Malik reported that PTTG1 is associated with cell angiogenesis, migration and invasion. PTTG1 in fact induces expression and secretion of MMP-2 (26). The functional blocking of PTTG1 may induce suppression of tumor growth and metastasis development, by the down-regulation of MMP-2 expression. Moreover, it is known that PTTG1 over-expression is associated with the secretion and expression of MMP-2. Previous data in HUVEC cells have been reported demonstrating that MMP-2 regulates cell migration, invasion, and endothelial tubule formation. All these data may bring to the conclusion that PTTG1 serves as one of the principal controller of MMP-2 and that some of the oncogenic effects of PTTG1 are mediated through the regulation of expression of MMP-2 (29). In neoplastic cells the expression of PTTG1 localized in the intertubular spaces, associated with OCT4 and KLF4 expression, suggest us that PTTG1 mark a specific subpopulation of SSC, characterized by high invasivity. The expression in seminoma stem cells of PTTG1 may permit them to invade surrounding tissues and to lead to neoplastic angiogenesis.

This study presents some limitations, which are represented by the limited number of clinical cases investigated and the absence of control cases (i.e., non-germ cell tumors).

## Conclusions

This pilot study provides the first identification of a cell population in seminoma characterized for being OCT4, KLF4, and PTTG1 positive cells in seminoma, associated with cancer invasiveness. Further investigation are needed to extend the number of clinical cases investigated, to analyze the co-localization of PTTG1 with MMP2, MMP9, VEGF and to clarify if a functional abrogation of PTTG1 might represent a novel therapeutic approaches in the clinical management of seminoma.

## Data Availability

All datasets for this study are included in the manuscript/supplementary files.

## Ethics Statement

Institutional Scientific Board of International Scientific Institute Paul VI approved the study. This study was conducted in accordance with the guidelines of the Declaration of Helsinki. Informed consent was obtained from each patient.

## Author Contributions

DM and FP: conceptualization. DM and GGr: literature analysis. MM, TC, and FM performed immunofluorescence analysis. DM and GGr: writing—original draft preparation. GGu and AB: writing—review and editing. AP: supervision.

### Conflict of Interest Statement

The authors declare that the research was conducted in the absence of any commercial or financial relationships that could be construed as a potential conflict of interest.
